# Biomechanical properties of adjustable extracortical graft fixations in ACL reconstruction

**DOI:** 10.1186/s40634-018-0154-4

**Published:** 2018-09-29

**Authors:** M. Ettinger, R. Karkosch, H. Horstmann, P. Savov, T. Calliess, T. Smith, M. Petri, the ESSKA under 45-committee (2016-2018)

**Affiliations:** Orthopaedic Surgery Department, Hannover Medical School (MHH), Anna-von-Borries-Str. 1-7, D-30625 Hannover, Germany

**Keywords:** Anterior cruciate ligament, Anterior cruciate ligament reconstruction, Tibial fixation, Extracortical graft fixation, Biomechanics

## Abstract

**Background:**

Reliable biomechanical data about the strength of different tibial extracortical graft fixation devices is sparse. This biomechanical study compares the properties of tibial graft fixation in ACL reconstruction with either the ACL Tight Rope™ or the Rigid Loop Adjustable™ device. The hypothesis was that both fixation devices would provide comparable results concerning gap formation during cyclic loading and ultimate failure load.

**Methods:**

Sixteen sawbone tibiae (Sawbones™) underwent extracortical fixation of porcine flexor digitorum profundus grafts for ACL reconstruction. Either the ACL Tight Rope™ (Arthrex) or the Rigid Loop Adjustable™ (DePuy Mitek) fixation device were used, resulting in 2 groups with 8 specimens per group. Biomechanical analysis included pretensioning the constructs 10 times with 0.75 Hz, then cyclic loading of 1,000 position-controlled cycles and 1,000 force-controlled cycles applied with a servohydraulic testing machine. Elongation during cyclic loading was recorded. After this, ultimate failure load and failure mode analysis were performed.

**Results:**

No statistically significant difference could be noted between the groups regarding gap formation during cyclic loading (4.6 ± 2.6 mm for the Rigid Loop Adjustable™ vs. 6.6 ± 1.5 mm for the ACL Tight Rope™ (*p* > 0.05)), and ultimate failure loads (980 ± 101.9 N for the Rigid Loop Adjustable™ vs. 861 ± 115 N ACL Tight Rope™ (*p* > 0.05)).

**Conclusion:**

ACL Tight Rope™ and the Rigid Loop Adjustable™ fixation devices yield comparable biomechanical results for tibial extracortical graft fixation in ACL reconstruction. These findings may be of relevance for the future surgical decision-making in ACL reconstruction. Randomized controlled clinical trials comparing both fixation devices are desirable for the future.

## Background

The ideal choice of graft fixation device in ACL reconstruction remains a matter of debate.

While interference screw fixation can provide good bony ingrowth of the graft, which can approximate the strength of the native ACL insertion site (Weiler et al., 2002a), several drawbacks such as tunnel enlargement and intraarticular screw prominence apply.

As an alternative, extracortical graft fixation devices can be utilized. Such extracortical fixation buttons have shown both promising biomechanical results in vitro (Johnson et al., 2015; Noonan et al., 2016; Petre et al., 2013) as well as good clinical outcomes (Boyle et al., 2015). Among extracortical fixation buttons, fixed-loop and adjustable-loop suspension devices are available. Adjustable-loop devices offer the opportunity of retensioning the graft while already placed in the tunnel. On the other hand, it has been advocated that fixed-loop fixation buttons would provide stronger biomechanical properties (Petre et al., 2013; Barrow et al., 2014).

With current early rehabilitation protocols following ACL reconstruction subject the graft construct to higher forces than what has been previously tested biomechanically, Johnson et al. biomechanically compared fixed-loop and adjustable-loop cortical suspension devices under high loads. They found lower cumulative peak cyclic displacement in fixed-loop devices compared to adjustable-loop buttons, with no significant difference in biomechanical properties after retensioning in adjustable-loop devices (Johnson et al., 2015).

Noonan et al. biomechanically evaluated adjustable loop devices, revealing that retensioning and knot tying after initial reduction of the tendon graft with an adjustable loop reduced final cyclic elongation by 50% when compared with a fixed-loop device (Noonan et al., 2016).

These somewhat contradictory previous studies reveal that reliable biomechanical data about the strength of adjustable extracortical tendon graft fixation devices is sparse.

Therefore, this study compares the biomechanical properties of 2 different adjustable extracortical fixation devices in ACL reconstruction using porcine flexor digitorum profundus tendon grafts and tibiae sawbones. The hypothesis was that both tibial extracortical fixation devices would provide comparable results concerning gap formation during cyclic loading and ultimate failure load.

## Methods

Being a biomechanical study using sawbone and porcine flexor tendons, no approval by the local ethics committee was necessary.

A total of 16 sawbone tibiae (Sawbones™ Europe, Malmö, Sweden) were used in this study. The proximal part of the sawbone was embedded in Polymethylmethacrylate (PMMA), with an additional long bicortical screw providing rotational stability within the potting.

Porcine flexor tendons derived from 12 months old fully-grown female or castrated male pigs were used in this study. The tendons were obtained from an industrial slaughterhouse and stored at − 20 °C. Before use, specimens were thawed at room temperature for 24 h. All tissues were kept moist using saline spray throughout the preparation and testing procedures.

Porcine tendon grafts were prepared to a length of 80 mm and a diameter of 10 mm. Tibial tunnels for ACL graft fixation were drilled in the anatomic position. The diameter of the tunnel was chosen equally to the diameter of the graft.

**Fig. 1:**
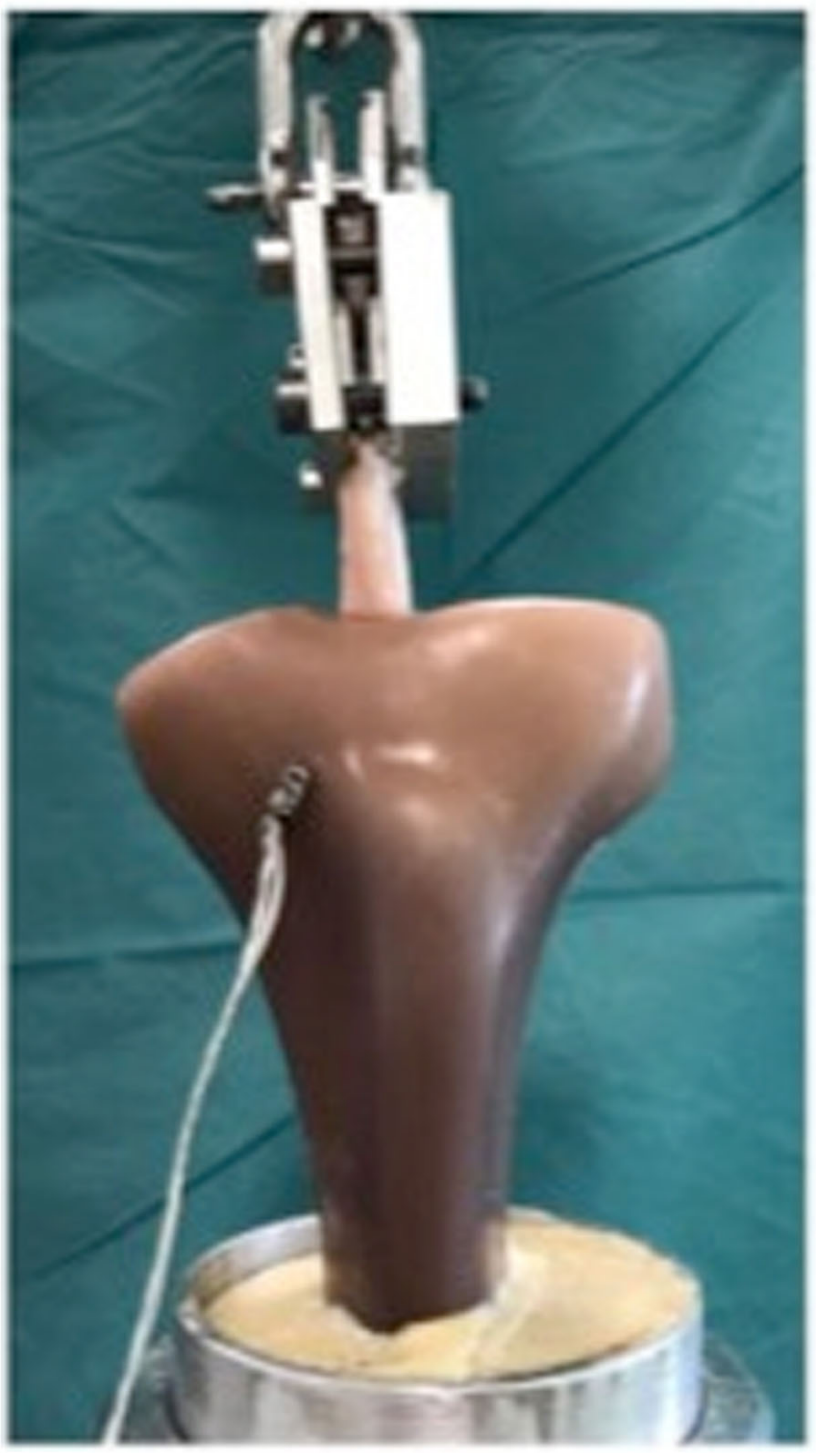
Extracortical tibial fixation devices were used in this study. Porcine tendons were mounted onto sawbone™ tibiae

Two different extracortical graft fixations were used for graft fixation (Fig. 1):

1. ACL Tight Rope™ (TR, Arthrex).
2. Rigid Loop Adjustable™ (RLA, DePuy Mitek).

Tendon grafts and fixations devices were randomly assigned to the sawbone tibiae.

### Biomechanical testing

The biomechanical setup was chosen according to previous studies of our research group (Ettinger et al., 2017).

Specimens were placed into a tensile loading fixation of a servohydraulic testing machine (Mini Bionix 858; MTS Systems Co, Minneapolis, MN, USA).

The tibia was fixed into a cup with screws so that the joint line was facing upwards. The proximal 20 mm of the tendon graft were rigidly fixed in a clamp in order to apply tensile forces on the fixation device, leaving 30 mm of distance between the tibial tunnel and the tendon clamp.

Preconditioning was done with 10 precycles with a retensioning on the tibial side before tibial knotting. All groups were then dynamically loaded for overall 2,000 cycles in position- and load-control mode each for 1,000 cycles at 0.75 Hz according to in-vitro loading parameters replicating the in-vivo ACL environment. During force-controlled cyclic loading loads between 10 N and 250 N were applied. Tensile load was applied in line with the tunnel axis along the ACL. Shear forces were not considered.

### Statistical analysis

All statistical analyses were performed using Statistical Package for Social Sciences (SPSS 15.0, SPSS Inc., Chicago, Illinois). Normal distribution was analyzed by the Shapiro-Wilk test. All values are presented in the form of mean ± standard deviation. Analysis of variance was used for parametric data and Kruskal-Wallis test for nonparametric data. A *p*-value < 0.05 was considered to be statistically significant.

A sample size calculation was performed using G*POWER (Heinrich-Heine-University, Düsseldorf, Germany). It revealed that a power of 0.95 would be achieved with a samples size for 3 for each group. With regard to other studies examining ACL properties with specimen this number was increased to 8 in order to respond to possible errors (Magen et al., 1999; Mayr et al., 2015).

## Results

**Fig. 2:**
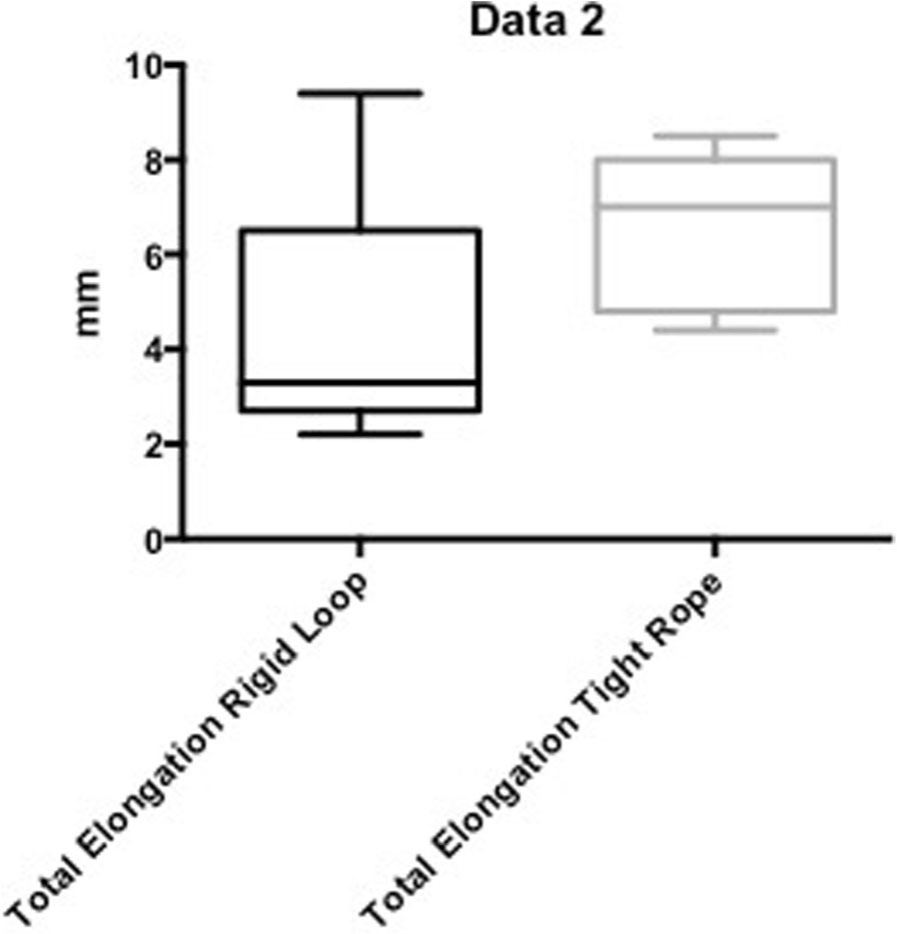
Results for gap formation (mm) during cyclic loading

**Fig. 3:**
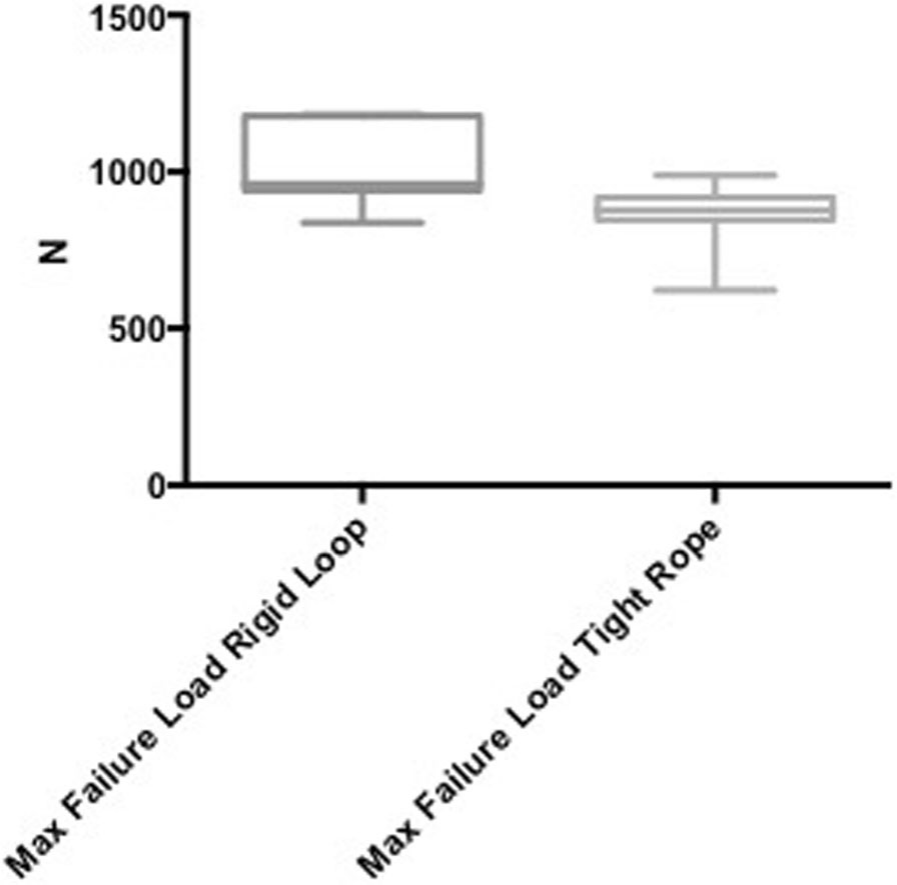
Results for ultimate failure load (N)

No statistically significant difference could be noted between testing groups regarding gap formation during cyclic loading (4.6 ± 2.6 mm for the Rigid Loop Adjustable™ vs. 6.6 ± 1.5 mm for the ACL Tight Rope™ (*p* > 0.05; Fig. 2)), and ultimate failure load (980 ± 101.9 N for the Rigid Loop Adjustable™ vs. 861 ± 115 N for the ACL Tight Rope™ (*p* > 0.05). Fig. 3 illustrates the median ultimate failure force required to induce failure. All suspensory devices withstood a force of 500 N before failure occurred.

Failure mode during ultimate failure load testing was a rupture of the graft in 8/8 cases in the Rigid Loop Adjustable™ group, and in 1/8 cases in the ACL Tight Rope™ group. Rupture of the knot occurred in 7/8 cases in the ACL Tight Rope™ group.

## Discussion

The most important finding of our study was that both tested extracortical fixation devices provided comparable biomechanical properties for tibial graft fixation.

Due to poor bone quality, the tibial graft side is referred to as “weak spot” in ACL-reconstruction (Brand Jr et al., 2000) emphasizing the need for improvement. Therefore our study focused on this aspect. Two suspensory devices were evaluated due to their capacity to withstand cyclic displacement and ultimate failure loads after ACL reconstruction in an biomechanical in vitro study.

It is widely accepted that elongation and ultimate failure force are crucial parameters for graft stability.

Ultimate failure loads have been reported as 1,725–2,160 N for the native ACL, 2,977 N for patellar tendon grafts, 2,352 N for quadriceps tendon grafts, and even 4,090 N for hamstring tendon grafts (West and Harner, 2005; Noyes et al., 1984). These numbers do not include any fixation of the tendon onto the bone, therefore emphasizing that the grafts themselves are even stronger than the native ACL, requiring to focus on graft fixation as the weaker spot of reconstruction.

However, these forces are applied to the whole ACL complex, there is no measurement for the tibial graft side, alone. In the present study we tried to limit this factor by using the identical setup on the femoral graft fixation side.

Graft fixation with interference screws can lead to a direct ligament insertion zone by creating compression of the graft against the bony tunnel walls. However, a variety of issues apply for interference screw fixation. As recommended by most manufacturers, the interference screw diameter should be similar or larger by + 1 mm for the tibial fixation in tendon grafts without bone blocks. Over dimensioned screws create strong initial compression, but can lead to eventual tunnel enlargement later on, creating difficulties in revision surgery (Buelow et al., 2002).

Bioabsorbable interference screws have been reported to be entirely absorbed and replaced by bone after around one to two years (Weiler et al., 2002a; Weiler et al., 2002b). Polylactid screws (Poly-L-Lactid, PLLA), which were used initially and thought to be absorbed over 3 to 5 years, were shown to sometimes not to get absorbed at all (Martinek et al., 2001; Stahelin et al., 1997). The ideal mixture of material is challenging, as absorption of the screw is a time-sensitive process.

As reported by Rodeo et al., extracortical graft fixation creates a fibrous layer between the tendon graft and the bone tunnel (Rodeo et al., 1993; Tomita et al., 2001). Studies from 1999 and 2000 have reported that this layer is later transformed into type II collagen and creates an indirect ligament insertion zone, which is due to longitudinal shearing instability, the so called “bungee effect” (Hoher et al., 1999; Jorgensen and Thomsen, 2000). In 2000, it was also reported that extracortical graft fixation has to deal with a long distance between the anchoring points of the graft, leading to elastic deformity of the construct, and eventually impeding bony ingrowth (Hoher et al., 2000).

On the other hand, recent studies investigating such extracortical fixation buttons have shown both promising biomechanical results in vitro (Johnson et al., 2015; Noonan et al., 2016; Petre et al., 2013) as well as good clinical outcomes (Boyle et al., 2015).

Among extracortical fixation buttons, fixed-loop and adjustable-loop suspension devices are available. In an effort to clinically investigate the suggestion that adjustable-loop graft suspension constructs in anterior cruciate ligament reconstruction may loosen after deployment, (Boyle et al., 2015) reported the two-year outcomes of their consecutive single-surgeon series of 188 patients with primary ACL reconstruction using hamstrings autografts. Seventy-three patients received adjustable-loop (TightRope RT, Arthrex Inc., Naples, FL) and 115 received fixed-loop (RetroButton, Arthrex Inc., Naples, FL) femoral cortical suspension. The authors found no significant difference between the two groups in KT-1000 testing at all time-points up to two years of follow-up. The rates of graft failure were similar too at 10% vs. 11%, respectively (Boyle et al., 2015). This study by Boyle et al. supports the clinical application of adjustable-loop suspension devices in ACL reconstruction.

The biomechanics of the two devices used in this study have been examined by Pasquali et al. in 2017 (Pasquali et al., 2017). They were able to show significantly higher forces for the RLA in comparison to TR under ultimate failure loads. Average displacement under cyclic loading was lower for the RLA (0.88 ± 0.14 mm vs. 1.13 ± 0.15 mm).

Our results regarding elongation under cyclic loading (4.6 ± 2.6 mm for the Rigid Loop Adjustable™ vs. 6.6 ± 1.5 mm for the ACL Tight Rope™, Fig. 3) exceed the suggested threshold of 3.0 mm displacement, defined as a clinical failure (Petre et al., 2013). This might be due to the fact that human hamstring tendons yield significantly lower initial elongation during preloading compared to porcine flexor digitorum profundus tendons as reported by Omar et al. (Omar et al., 2016). However, this study also found that biomechanical properties during cyclical loading were comparable. Additionally, they found that human hamstring tendons also showed significantly higher maximum failure loads than porcine flexor digitorum profundus tendons (1597 ± 179.6 N vs. 1109 ± 101.9 N; *p* = 0.035) (Omar et al., 2016). Both, the higher initial elongation during preloading and lower maximum failure load of porcine flexor digitorum profundus tendons needs to be taken into consideration for this study. However, taking into account the ultimate failure loads of fixation devices as shown in our study (980 ± 101.9 N vs. 861 ± 115 N, respectively), the ultimate failure loads of porcine flexor digitorum profundus tendons still are higher.

Furthermore, the loading value of the ACL during daily activities has been reported up to a maximal value of approximately 454 N (Noyes et al., 1984). The major advantages of porcine flexor tendons include good availability, and lower biomechanical variability compared to human cadaveric specimens.

The properties of sawbone femura compared to human cadaveric femura have been investigated in previous studies (Heiner, 2008; Gardner et al., 2010). Similar to porcine tendons, the advantages of sawbones include good availability, and lower biomechanical variability compared to human cadaveric specimens. Results of published cadaveric biomechanical studies are oftentimes spread over a broad range, which is most likely due to the anatomic variability among cadaveric specimens. Composite analogue bone models such as sawbone are able to mimic the structural properties of average healthy adult human bones (Gardner et al., 2010).

Several limitations apply to this study. First, with 8 specimens per group, the sample size of this study was limited. Second, this controlled laboratory study reflects the mechanical properties of femoral ACL fixations without any biological healing or remodeling responses. Third, as outlined above, the applicability of sawbones and porcine flexor digitorum profundus tendons needs to be thoroughly reflected. Due to the setup shear forces could not be considered. In vivo studies are desirable to further investigate the biological behaviour in the future.

## Conclusion

ACL Tight Rope™ and the Rigid Loop Adjustable™ fixation devices yield comparable biomechanical results for tibial extracortical graft fixation in ACL reconstruction.
